# Supplementary material for: Higher-Order Multiphoton Absorption Upconversion Lasing Based on ZnO/ZnMgO Multiple Quantum Wells
Source: Nanomaterials (Basel). 2022 Sep 4;12(17):3073. doi: 10.3390/nano12173073 (PMC9457886; doi:10.3390/nano12173073)
Supplement: Supplementary file 1 [file nanomaterials-12-03073-s001.zip › nanomaterials-1838345-supplementary.pdf]

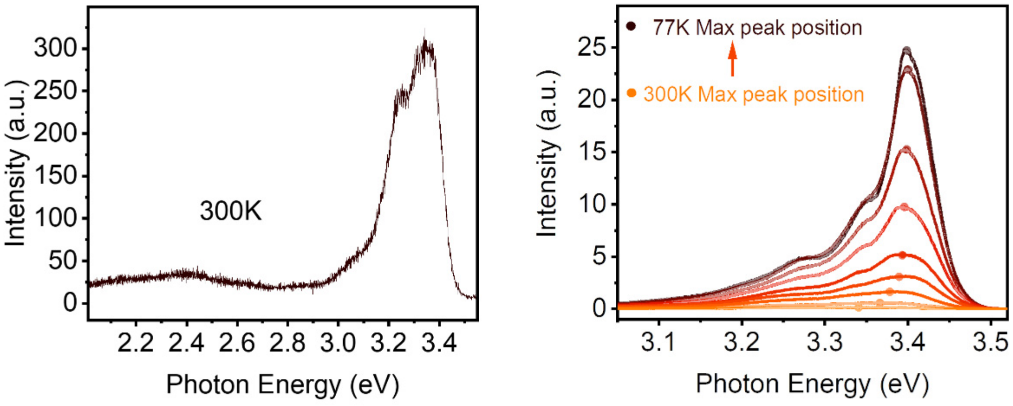

(a) (b)

Figure S1. (**a**) The PL spectra of the MQWs at 300 K. (**b**) The maximum peak position of PL spectra of the MQWs at different temperatures.

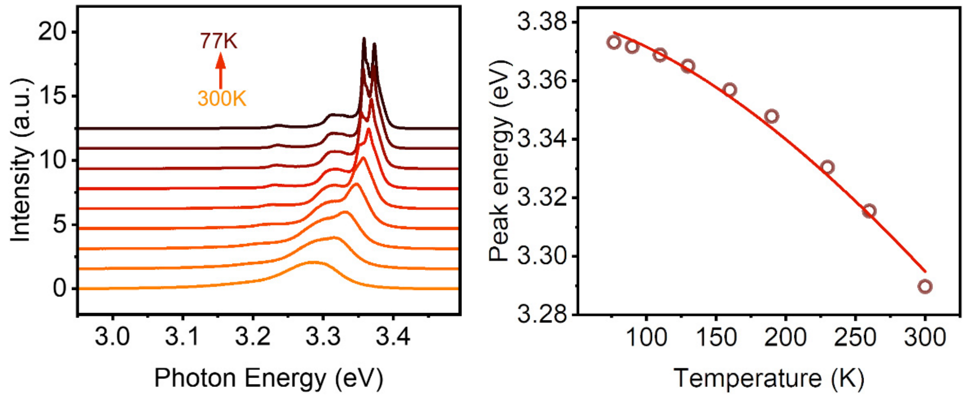

(**a**) (**b**)

Figure S2. (**a**) The temperature-dependent PL spectra of the ZnO film from 77 to 300 K. (**b**) The PL peak position with temperature for the ZnO film. Red solid lines show the fits to the experimental data.

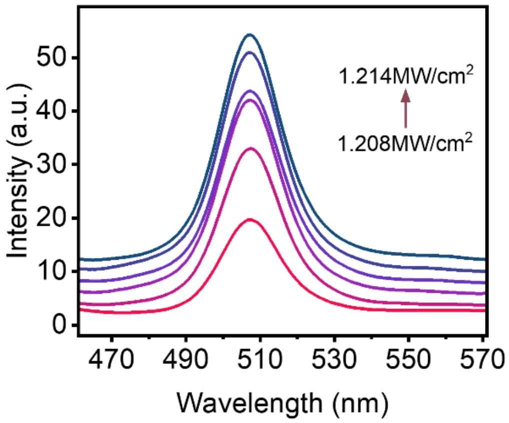

Figure S3. The variation diagram of the third harmonic peak with power density.

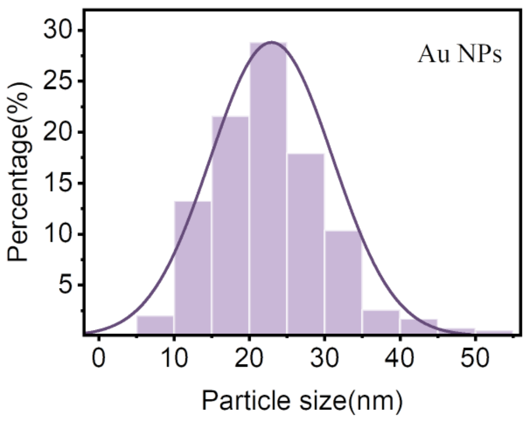

Figure S4. The histogram of size distribution of Au NPs.
